# Anthocyanin‐loaded bacterial cellulose nanofiber as a green sensor for monitoring the selective naked eye and visual detection of Al(III) Ions

**DOI:** 10.1002/ansa.202300014

**Published:** 2023-05-10

**Authors:** Sima Arghavani, Fatemeh S. Mohseni‐Shahri, Farid Moeinpour

**Affiliations:** ^1^ Department of Chemistry, Bandar Abbas Branch Islamic Azad University Bandar Abbas Iran

**Keywords:** aluminium ion, anthocyanin, bacterial cellulose nanofiber, green, metallochromic sensor

## Abstract

The present study developed a green metallochromic sensor that detects aluminium (Al(III)) ions in solution and solid state using anthocyanin extract from purple onion peels embedded in bacterial cellulose nanofibers (BCNFs). The CIE Lab colour parameters demonstrated that Al(III) binding causes a sensible change in colour. A variety of metal ions including K^+^, Mn^2+^, Cu^2+^, Hg^2+^, Cr^2+^, Pb^2+^ and Ni^2+^ were used to challenge the sensor to determine its selectivity. The findings demonstrated that the suggested sensor showed excellent selectivity toward Al(III) ion. Al(III) is quantitatively detected by the sensing method with detection limits in the range between 30–200 and 20–300 ppm in solution and solid state, respectively, and through observation with naked eye. The fabricated green metallochromic sensor is promising to be a simple, cheap, mobile and easily operable for real‐time and on‐site detection of Al(III) ions in food matrices.

**Abbreviations**
BCNF: Bacterial cellulose nanofibers
Al(III): Aluminum ion
BCNF‐ANT: anthocyanin bacterial cellulose nanofiber
BCNF‐ANT‐Al: anthocyanin bacterial cellulose nanofiber after the adsorption of Al(III)
CNC: Cellulose nanocrystals
CNFs: cellulose nanofibers

## 1 INTRODUCTION

Among the elements in the earth's crust, aluminium ranks third in terms of abundance after oxygen and silicon, which is used in food additives, building materials and kitchenware. Aluminium has long been considered harmless to humans but has been identified as a toxic metal for over a hundred years.^1^ Recently, several studies have shown that aluminium can damage many parts and cells of the human body, such as the brain, bones and blood, and has been linked to illnesses such as Alzheimer's, anaemia, osteoporosis, Shaver's disease, Parkinson's disease and even lung and breast cancer.^2^, ^3^, ^4^, ^5^ The mouth, drinking water, food, medicine and the environment are the sources that allow aluminium to enter the human body. It is urgently necessary to develop highly sensitive and selective chemical sensors for detecting Al(III) ions due to the potential negative impacts that they can have on human health and the environment.^6^ Thus, it is important to identify and control the concentration of Al(III) ions. However, so far, few chemical sensors have been identified to detect Al(III) ions.

It has been discovered that Al(III) ions can be detected using several analytical techniques, such as inductively coupled plasma optical emission spectrometry, flame atomic absorption spectrometry and inductive coupled plasma mass spectroscopy.^7^, ^8^, ^9^ There are limitations to the applications of the mentioned techniques because they involve highly developed equipment, time expenditure, sample destruction methods and the costly and tedious process of sample preparation. Colorimetric sensors have received considerable attention due to their simplicity and capabilities of on‐site monitoring, among the diverse detection techniques.

In recent years, various compounds have been used for the colorimetric sensing of Al(III) ions to include azo dyes, rhodamine, photochromic diaryl ethylene, naphthyridine and Schiff bases derived from 2‐hydroxybenzaldehydes.^10^, ^11^, ^12^ Contrary to their efficacy, suchlike compounds are toxic. Therefore, search for eco‐friendly sensor molecules, such as natural pigment, is vital.

**FIGURE 1:**
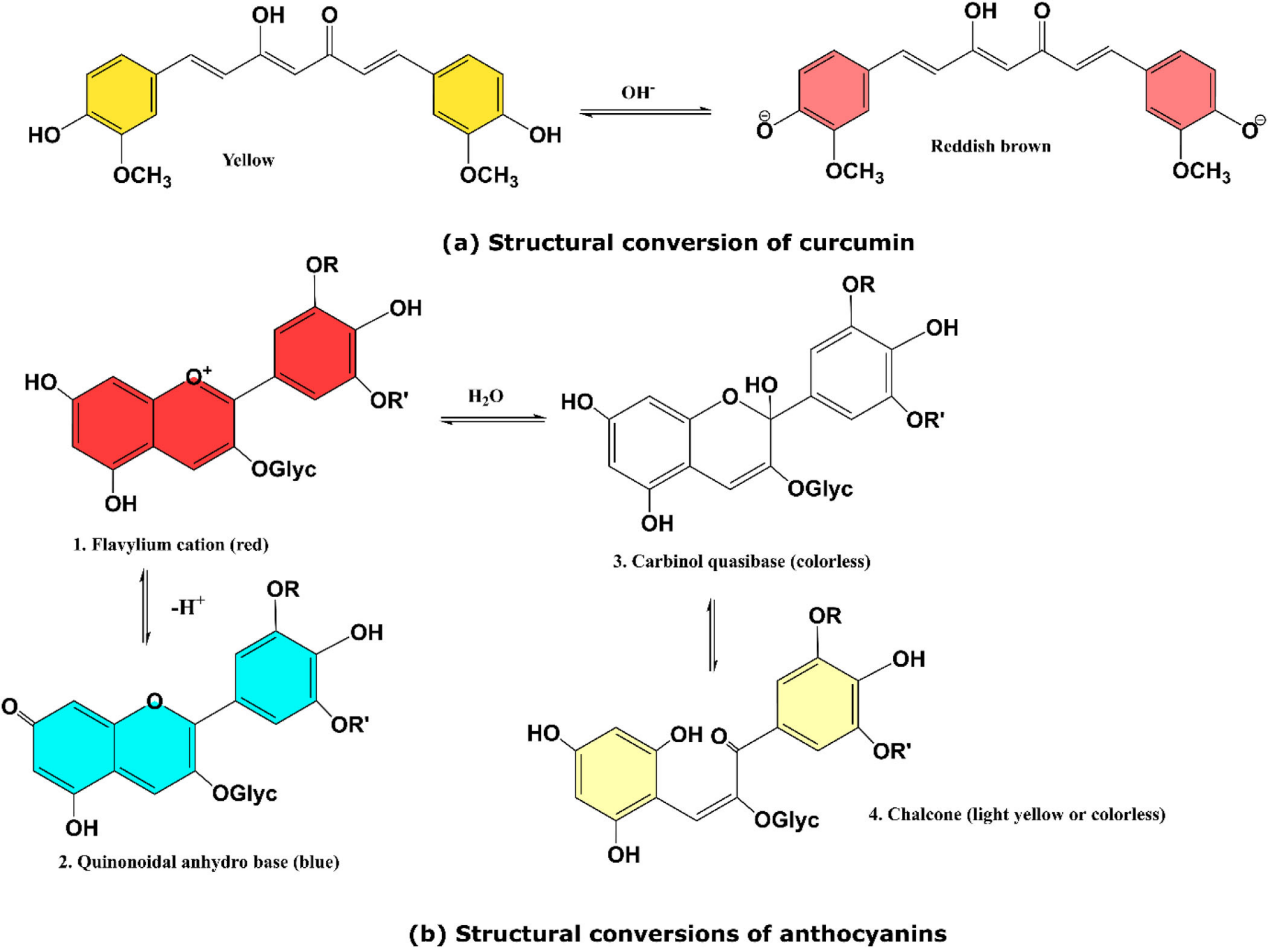
Structural transformation of anthocyanin in various pHs.

In plants, fruits and vegetables, water‐soluble pigments known as anthocyanins give these products their red, blue or purple colour.^13^ They also belong to a class of compounds called flavonoids, which have antioxidant properties and have drawn significant interest in the field of food science as a result of the health benefits they provide.^14^ Many conjugated phenolic rings are found in anthocyanins. Anthocyanin molecules undergo protonation and deprotonation along the phenolic rings along which they are comprised, and this alters the pattern of conjugation throughout the molecule, which leads to changes in colour as a result (Figure 1).^15^ In low pH, anthocyanin tends to be red, in neutral pH, it is blue/purple and in high pH, it is green/yellow.

Furthermore, anthocyanins have shown biological activity, like antioxidants and anti‐cancers, primarily because they quench reactive oxygen species.^14^ Organic food waste such as red cabbage, radish, black carrots, grapes, purple onions and other coloured foods contain anthocyanins. There are a wide variety of vegetables in the world today, but purple onions are among the more common. The dyeing of fabrics has previously been accomplished with anthocyanins found in purple onion peel. However, they have not been employed in chemical sensors for sensing of metal ions.^16^ Cellulose is the most abundant biopolymer in nature.^17^ Depending on the morphology, methods and functions of cellulose, three main categories can be described as follows: Cellulose nanocrystals (CNCs), cellulose nanofibers (CNFs) and bacterial cellulose nanofibers (BCNFs). BCNFs produced by non‐pathogenic bacteria *Acetobacter xylinum* as promising biomaterials have shown great potential in developing paper sensors. A bacterial nano‐cellulose paper has been selected as an effective substrate for the development of a new generation of paper‐based sensors due to its outstanding mechanical and chemical properties, excellent biological properties (biocompatibility, environmental degradation), porous matrix, optical transparency and printability.^18^ There are a variety of optical nanoparticles, luminescence or colorimeters that can be embedded into the three‐dimensional, porous BCNF nano‐network scaffold.^19^ As a low‐cost green material, BCNF can act as a substrate combined with active materials, widely used in sensor and energy storage‐related areas.^20^ The main advantages of BCNF as a substrate are renewability, biodegradability, biocompatibility and possession of shape flexibility, mechanical strength, large specific surface area and ultrafine fibre.^21^, ^22^ In addition, the porous network structure is conducive to the infiltration and combination of active materials on BCNF to form functional sensing materials, broadening the application of BCNF and providing a new view for developing flexible sensors. On the one hand, the orderly stacked crystalline regions formed by abundant hydrogen bonds endow BCNF with excellent mechanical properties.^23^ On the other hand, strong intramolecular and intermolecular hydrogen bonds make the network structure of BCNF quite tight and difficult to dissolve.^24^ Therefore, there is limited development for the exploitation of BC‐based functional sensing materials, and available fabrication strategies are needed to design and develop BCNF‐based sensing materials. The development of cellulose‐based sensors is attracting wide and increasing attention, and cellulose has been adopted for construct physical, bio and chemical sensors individually or in combination with other materials.^25^

Next to water, tea is the most widely consumed beverage in the world.^26^ The tea plant is able to absorb relatively large amounts of essential elements for the body such as chromium and cobalt^27^ as well as unnecessary and harmful elements such as aluminium, arsenic and cadmium from the soil and store them in its leaves.^28^ Tea is a major dietary source of Al(III), and tea drinking can more than double an individual's intake of Al(III). Therefore, drinking contaminated tea can cause health risks. Examining the nutritional composition of tea, especially essential, non‐essential and toxic elements in terms of quality, nutritional aspects, health and pollution is of great importance.^29^ The measurement of Al(III) in tea can determine the amount of this element entering the body through tea compared to the total daily amount of its intake.

Based on the interactions between anthocyanin phenolic dye and Al(III) in the solution state and the BCNF sheet's 3D nano‐network scaffold, the present study developed a biocompatible and simple sensor displaying colour changes when exposed to Al(III) ions. Both selectivity and detection limits were described. In this platform, detecting Al(III) ions can be done efficiently, specifically, quickly, easily and at an affordable price.

## 2 MATERIAL AND METHODS

### 2.1 Materials

Nano Novin Polymer Co. (Sari, Iran) provided wet BCNF sheets for the study. From the local market, purple onion was purchased (Bandar Abbas, Iran).

Salts of metal, including Pb(NO_3_)_2_, KNO_3_, Cu(NO_3_)_2_, Hg(NO_3_)_2_, CrCl_3_, AlCl_3_, NiCl_2_ and MnSO_4_, were supplied from Merck. Suitable amounts of these salts were dissolved in distilled water to prepare stock aqueous solutions. At first, the aluminium (III) chloride stock solution was prepared at 1000 ppm, followed by serial dilutions (100–1000 ppm). Acetate buffer solutions were prepared in the laboratory using sodium acetate and glacial acetic acid. The rest of the reagents (analytical grade) were provided from Sigma‐Aldrich.

### 2.2 Extraction of anthocyanin

Anthocyanin used in preparation of the films was extracted from the purple onion peel according to the methodology described by Liang et al.^30^ In order to powder purple onions, the peels of the purple onions were peeled and dried at room temperature. In a solution of 40% (v/v) ethanol with pH adjusted to 2.0 by 1 mol/L HCl, 100 g of purple onion peel powder was immersed. It was then filtered to remove solids after 24 h in a refrigerator in the dark. For further analysis, the extract was placed in a refrigerator at a temperature of 4^◦^C.

### 2.3 Instruments

The absorbance was measured at 300 and 700 nm using a UV–Vis spectrophotometer (SPEKOL 1500). ATR‐FTIR spectra were recorded using a Thermo Nicolet 370 (Thermo Fisher, the USA). The spectra were registered at 2 cm^−1^ between 4000 and 400 cm^−1^. Field emission scanning electron microscopy (FE‐SEM) (TESCAN, Czech Republic) was applied to acquire the SEM images.

### 2.4 Characterisation of anthocyanin

A flavonoid‐characteristic test was conducted using Shinoda's approach. Using a piece of magnesium ribbon and hydrochloric acid (1 mL), a Shinoda test was carried out to determine whether the anthocyanin extract generated a red colour once added to the methanolic extract (2 to 3 mL).^31^

### 2.5 Provision of BCNF‐ANT

For 90 min at 25°C under gentle shaking (50 rotations per minute), five pieces of BCNF sheets (20 × 2.5 × 0.3 cm^3^) were soaked in 100 mL of anthocyanin solution. To remove the un‐trapped anthocyanins from the anthocyanin bacterial cellulose nanofiber (BCNF‐ANT) sheets after the sheets were separated from the solution, several washes with deionized water were performed to remove the anthocyanins from the sheets. Binder clips were used to sandwich the BCNF‐ANTs between two glass slides covered with regular filter paper (Whatman). After being kept at 100°C for 2 h, the glass slides were removed from the oven. To apply the BCNF‐ANTs, the dried BCNF‐ANTs were stored in a brown, dry container.

### 2.6 Analysis of the Al(III) ions by colorimetry

Using anthocyanin extracted solution and generated BCNF‐ANT film at ambient temperature, the presence of Al(III) was monitored. At a neutral pH value of ∼7.0, aluminium chloride at different concentrations ranging from 10 to 1000 ppm was dissolved in distilled water. The 50 µL of anthocyanin was added to each of the aluminium solutions at different concentrations. Aqueous solutions containing other metal salts, including Pb(NO_3_)_2_, KNO_3_, Cu(NO_3_)_2_, Hg(NO_3_)_2_, CrCl_3_, AlCl_3_, NiCl_2_ and MnSO_4_, have also been prepared and tested under the 200 ppm concentration. Different coloration measurements were taken after immersing the BCNF‐ANT sensor film in each aqueous solution.

### 2.7 Coloration measurements

A freeware version of ImageJ^®^ (http://imagej.nih.gov/ij) was used to monitor the colorimetric changes under parameters established by the CIELAB. The Δ*E* values were calculated as follows:^32^

(1) $$\Delta E=\sqrt{{\left(L^{*}-L_{0}^{*}\right)}^{2}+{\left(a^{*}-a_{0}^{*}\right)}^{2}+{\left(b^{*}-b_{0}^{*}\right)}^{2}},$$

where, ${L_{0}}^{*},{a_{0}}^{*}$ and ${b_{0}}^{*}$ were the numerical values of lightness (white to black), colour ranges from red to green and colour ranges from yellow to blue, respectively, gathered at 0 min, and L^*^, a^*^ and b^*^ were the values at the time for sampling.

### 2.8 Determination of Al(III) in tea

An accurate weight (4.0 g) was applied to a crucible. Six millilitre of concentrated hydrochloric acid and 2 mL of concentrated nitric acid and were added. In 25 min, a mild heat treatment was applied to the sample to digest the contents till they were nearly dry. After that, it was ashed at 600°C for 1 h in a muffle furnace. It was then evaporated to near dryness with 5 mL of nitric acid (1:1, v/v). For ashing, 2 g of ammonium peroxydisulfate was added and the sample was put in a muffle furnace at 800°C for 1 h. It was taken out and cooled. To dissolve the residue, 10 mL of HNO_3_ (1: 99, (v/v)) was added. Acetate buffer solution was used to dilute the sample to 100 mL.

## 3 RESULTS AND DISCUSSION

### 3.1 Characterisation of anthocyanin and BCNF‑ANT films

It was determined by Shinoda's test whether flavonoids were present in the product and a red colour in a few minutes was produced.

#### 3.1.1 ATR‐FTIR spectroscopic investigation

**FIGURE 2:**
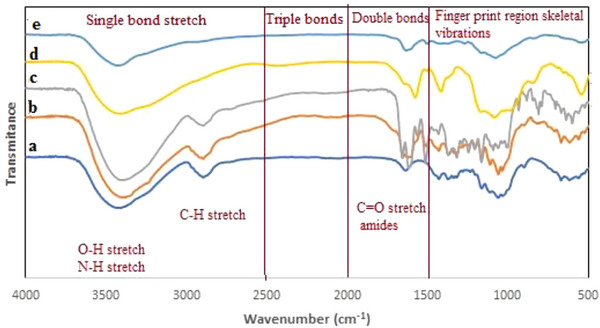
ATR‐FTIR spectrum of (a) BCNF, (b) BCNF‐ANT, (c) BCNF‐ANT‐Al(III), (d) diluted ANT and (e) diluted ANT‐Al(III).

As a result of using ATR‐FTIR spectroscopy, the chemical structure of the biopolymer/polymer materials was determined, as well as the presence or absence of specific functional groups. There are two kinds of functional groups: hydroxyl and amine. Interactions with other substances can alter these functional groups' vibrations (and spectral position).^33^ Figure 2 shows the ATR‐FTIR spectra of BCNF, BCNF‐ANT, BCNF‐ANT‐Al (after the adsorption of Al(III) ion), diluted ANT and diluted ANT‐Al(III) (2a‐e), respectively.

BCNF has the following characteristic bands: 3341 cm^−1^ related to O‐H and N‐H tensile of the amide‐A group coupled to hydrogen,^34^ 2894 cm^−1^ related to C‐H tensile vibration and 1427, 1314, 1159, 1105 and 1031 cm^−1^ related to C‐C tensile vibration, skeletal vibrations and ring vibrations.

BCNF films contained anthocyanins, as indicated by the peaks associated with the stretching of aromatic rings (1600−1585; 1500‐1400 cm^−1^). C = O stretching of BCNF carboxyl groups explains the amide‐I band observed in samples at 1629 cm^−1^. Anthocyanin O–H groups formed hydrogen bonds with BCNF O–H and N–H groups as reflected in marked changes in peaks at ∼3500–3200 and ∼1650–1500 cm^−1^.^35^ An interaction between Al(III) and BCNF‐ANT resulted in a shift in the C = O peak at 1600 cm^−1^, which confirmed that the complex formation between anthocyanin and Al(III) ions involves C = O groups.^36^

The spectrum for the diluted ANT (Figure 2d) displays a distinguished absorption band at 1650 cm^−1^ related to the tensile vibration of the C = C aromatic ring. Comparing the spectra of diluted ANT‐Al(III) to diluted ANT, a more substantial impact is noted in the absorption band at 1650 cm^−1^ (Figure 2e). When Al(III) ions were added to the mixture, the intensity of the mentioned band decreased.

#### 3.1.2 Morphological properties of BCNF‑ANT films

**FIGURE 3:**
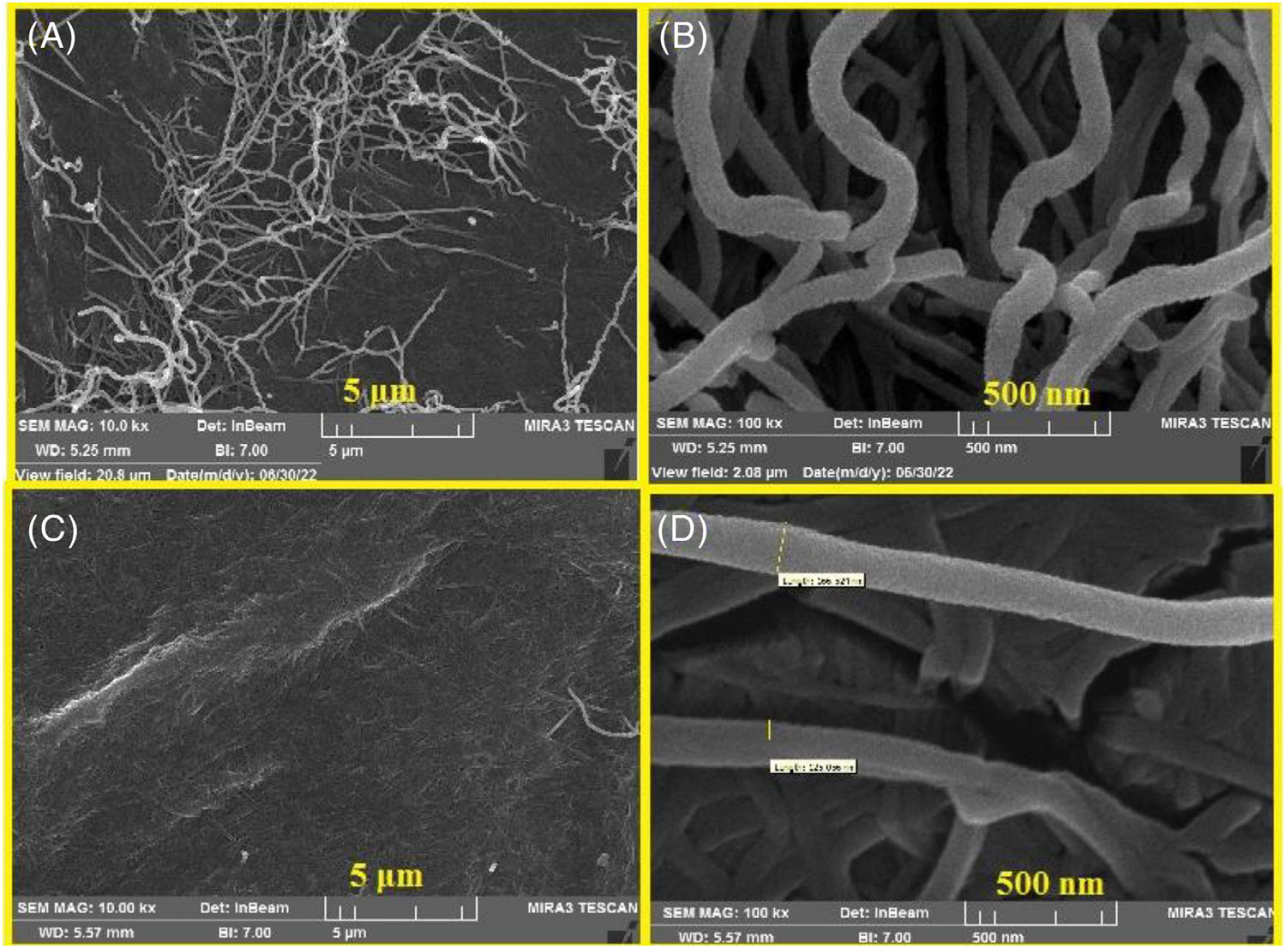
FESEM pictures of (a,b) BCNF and (c,d) BCNF‐ANT with a 5 µm and 500 nm scale bar.

The immobilisation of anthocyanin extract within the BCNF film was investigated by scanning electron microscope (SEM) images. According to the described protocol, Figure 3 shows BCNF and BCNF‐ANT SEM images. The difference between two SEM micrographs with a scale bar of 5 µm (Figures 3a and c) showed that anthocyanin was present and incorporated into the transparent nano‐network of BCNF. As shown in a scale bar of 500 nm, all two surfaces have nanofibrous miniaturised morphology of BCNF.

### 3.2 Colorimetric performance of anthocyanin solution and BCNF‑ANT film

**FIGURE 4:**
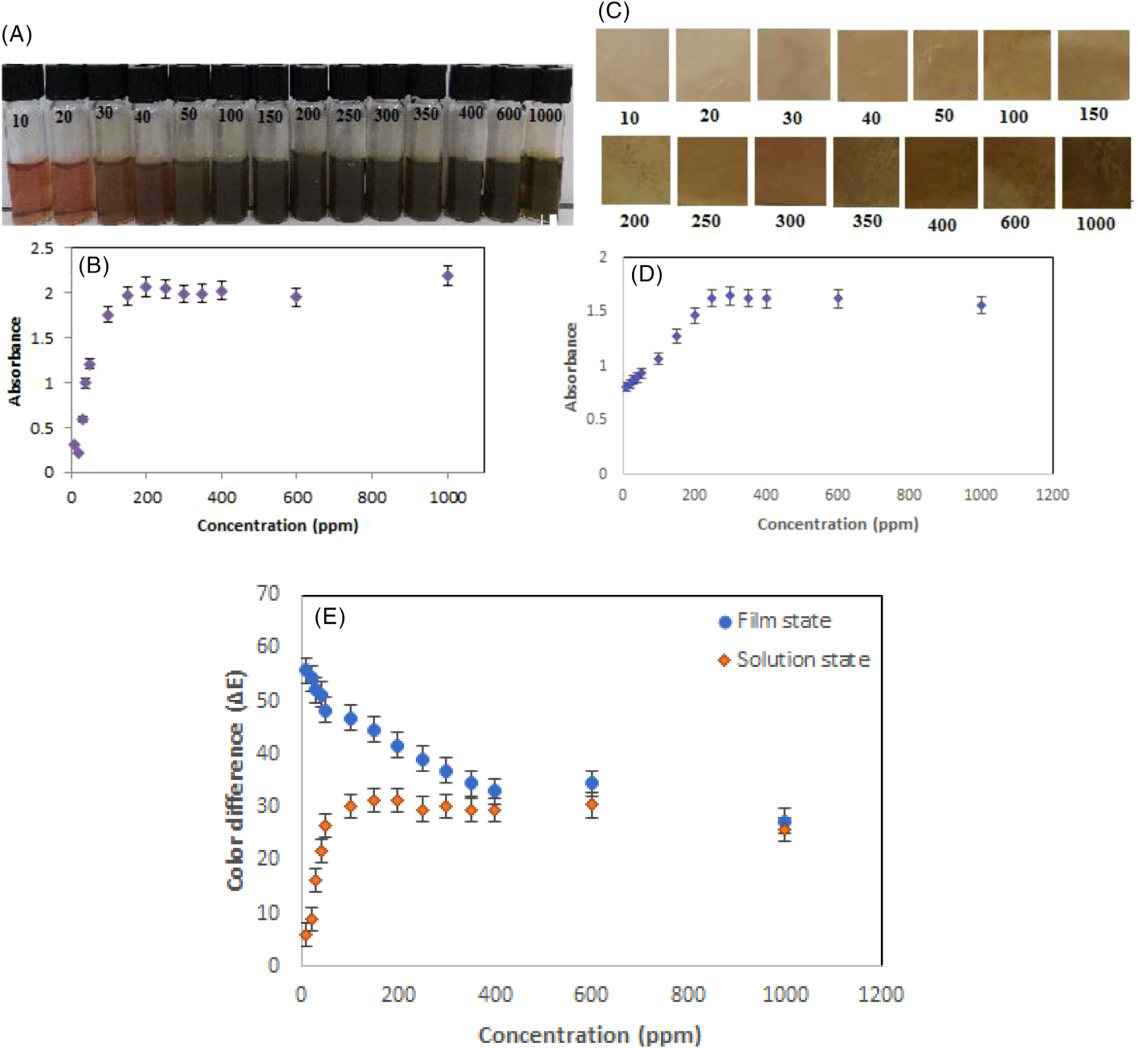
Color variation of (a) anthocyanin solution and (c) BCNF‐ANT sensor in different concentration (10–1000 ppm) of Al (III), changes in absorbance intensity of (b) anthocyanin solution and (d) BCNF‐ANT sensor at 485 nm upon sensing the increased concentration (10–1000 ppm) of Al (III) in aqueous medium, (e) total colour difference (Δ*E*) of anthocyanin solution and BCNF‐ANT sensor in different concentration (10–1000 ppm) of Al (III).

As shown in Figure 4(a) in the solution state with the increasing of the Al(III) ion concentration from 10 to 1000 ppm, visible colour changes ranging from pink to dark green.

There is evidence that the establishment of coordination bonds between phenolic anthocyanins and Al(III) leads to the formation of complexes of various degrees of green colour, within the context of the aluminium concentration. Al(III) aqueous total content showed a correlation profile (Figure 4b).

At 485 nm, the absorbance intensities showed a correlation profile directly proportional to the increase in Al(III) concentration between 10 and 1000 ppm; that is, the absorbance intensities increased as Al(III) concentration increased. Upon lowering the concentration of the aqueous Al(III) below 30 ppm, no changes in the absorption intensity of the solution at 485 nm were observed. As a result, less than 30 ppm was monitored as the detection limit. Within the increase of the Al(III) concentration, the visible colorimetric changes were detected. The findings indicated that by raising the Al(III) concentration to 200 ppm, no increase in the absorption peak intensity was observed at 485 nm, so the detection limit was determined by increasing the concentration of aluminium to reach 200 ppm.

According to Figure 4(c), the BCNF‐ANT tape can be discerned with visible colour when exposed to Al(III) concentrations ranging from 10 to 1000 ppm. The absorbance intensities at 485 nm are shown in Figure 4(d). It displayed a detection limit as low as 20 ppm and as high as 300 ppm.

It is important to note that the image analysis was conducted following Equation (1) and Δ*E* values represented in Figure 4(e). The results indicate that with enhancement of the Al (III), Δ*E* values of the solution state increased and in film state decreased.

### 3.3 Selectivity of anthocyanin solution and BCNF‑ANT film towards Al(III) ions

**FIGURE 5:**
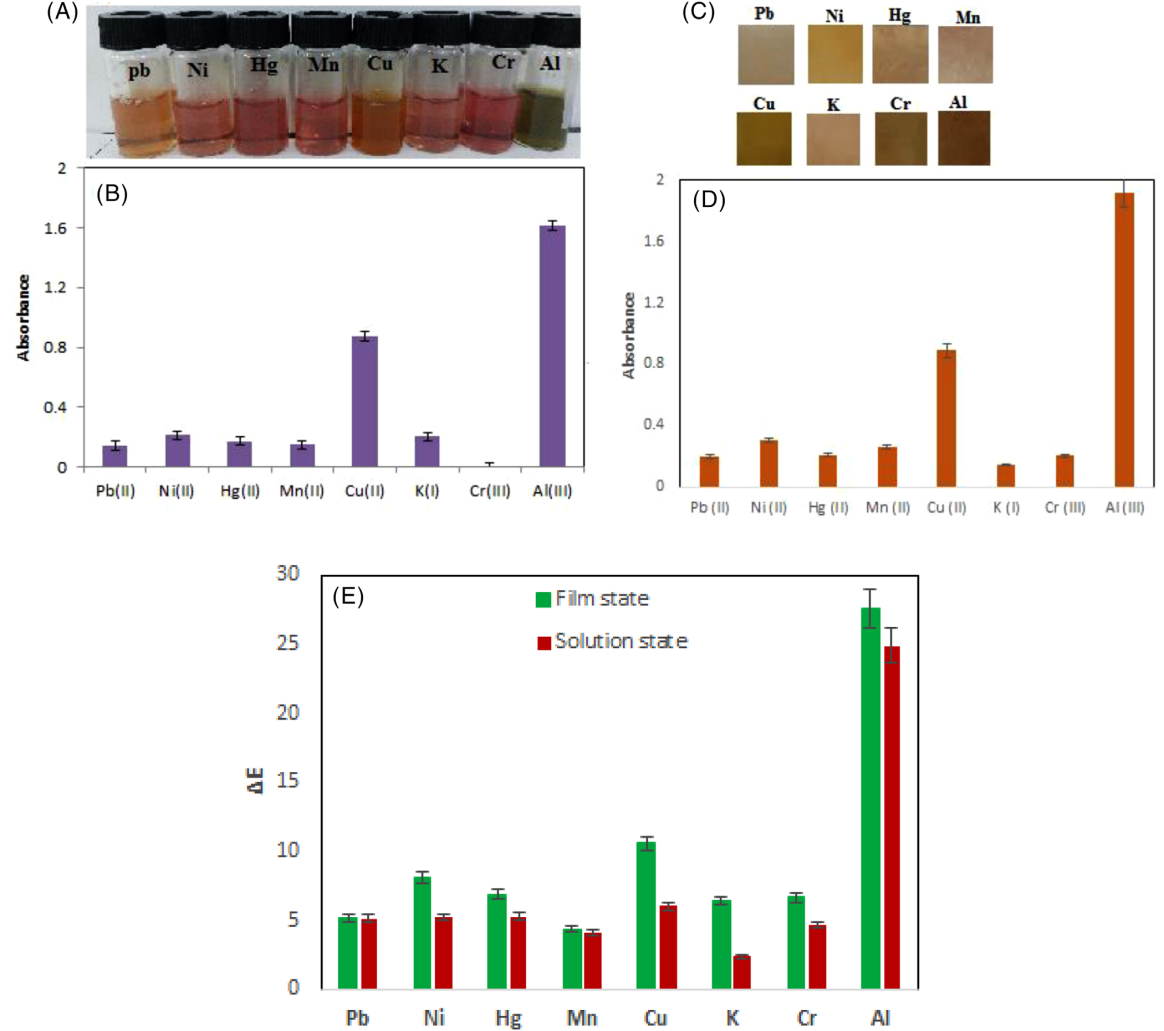
Selectivity evaluation of the (a) anthocyanin solution and (c) BCNF‐ANT sensor (200 ppm), comparative insight on the intensities of the absorption maxima for (b) anthocyanin solution and (d) BCNF‐ANT sensor at 485 nm for different metal cations (200 ppm), (e) the Δ*E* values for the various interference ions (200 ppm).

The selectivity of anthocyanin solution and BCNF‑ANT film towards Al(III) ions was studied in the presence of the various selected metal ions, containing K^+^, Mn^2+^, Cu^2+^, Hg^2+^, Cr^3+^, Pb^2+^ and Ni^2+^. As seen in the absorption spectra at 485 nm, the anthocyanin solution was highly selective towards Al(III) ions in water (Figure 5a). A comparative intuition on the absorption intensities of anthocyanin solution at 485 nm for the chosen metal ions (at constant concentration; 200 ppm) is shown in Figure 5(b).

As well, BCNF‐ANT sensor selectivity was investigated when metal ions were present. A total of 0.5 h of incubation was performed on the BCNF‐ANT sheets using 200 ppm of each ion. As shown in Figure 5(c), there was a noticeable colour difference between the chosen metal ions and Al(III). BCNF‐ANT changed colour because anthocyanin complexed with Al(III). The calculated Δ*E* values are indicated in Figure 5(e).

Nevertheless, Cu(II) displayed a low sensitivity to anthocyanin solution and BCNF‐ANT sensor like Al(III) ions [21]. In the presence of Al(III), the colour intensity of BCNF‐ANT sensor was higher than Cu(II). Also, the a* positive value decreased to display a colour shift from redder to greener shadows, while b* positive value decreased to ascertain a colour change from yellower to bluer shadows.^37^, ^38^, ^39^

### 3.4 Sensor sensitivity to pH

**FIGURE 6:**
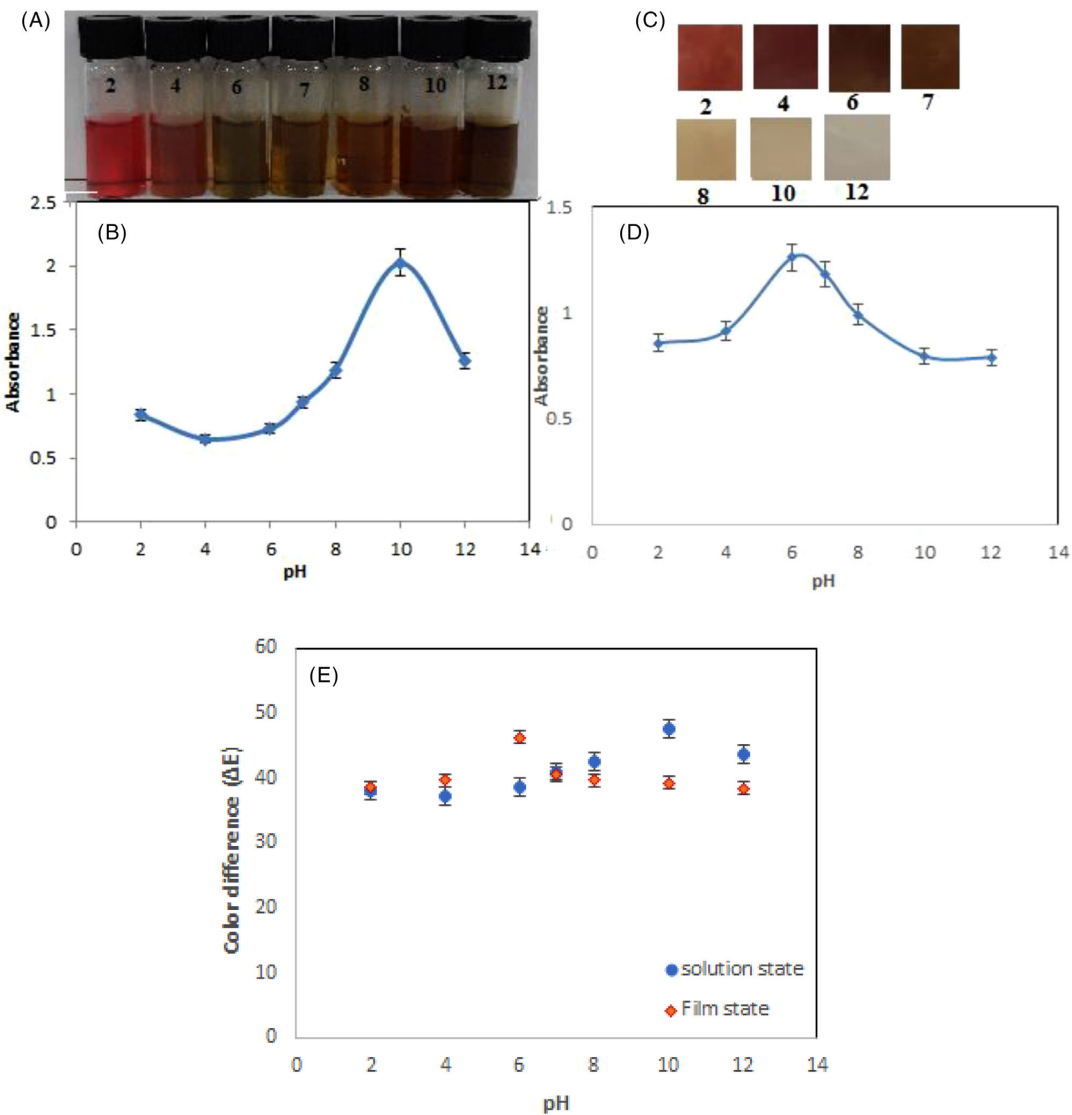
Colour variation of (a) anthocyanin solution and (c) BCNF‐ANT sensor (200 ppm) at different pH, changes in absorbance intensity of (b) anthocyanin solution and (d) BCNF‐ANT sensor at 485 nm upon sensing Al (III) (200 ppm) in aqueous media at various pHs, (e) the Δ*E* values in various pHs.

Changes in pH affect anthocyanins. So, 200 ppm Al(III) was prepared as a stock aqueous solution. Buffer solutions containing acetate were added to increase the pH value from 2 to 12. UV‐Vis spectra were recorded for the solutions prepared at different pH levels. Figure 6 (b,d) shows the graph of the UV‐Vis absorbance intensity versus the pH value in the solution and film state.

At pH less than 6, the changes in UV‐Vis absorbance intensity are insignificant, which can be due to the high coagulation of Al(III) ions in acidic pH. At pH between 6 and 10, the anthocyanin molecules act as a rigid anion and form a complex with the Al(III) cation.

At pH 7, the anthocyanin was negatively charged, and the aluminium was mainly in the form of Al(OH)_2_^+^ and Al(OH)_3_. At the same time, the biopolymers (BCNF) were deprotonated, leading to the electrostatic attraction between the film surface and the aluminium species, facilitating the formation of the ANT‐Al complex.^40^

The total colour change of the sensor was calculated by colour parameters of the sensor at pH 2‐12. As seen in Figure 6(e), the highest values of Δ*E* for solution and film state were recorded at pH 10 and 6, respectively. Also, Δ*E* values in other pHs were greater than 20.

### 3.5 Real sample measurement

**FIGURE 7:**
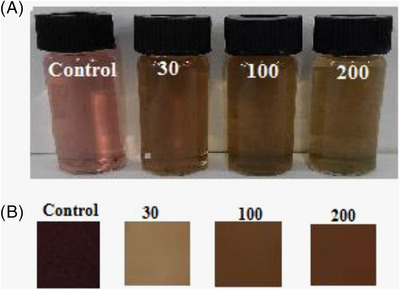
The pictures of the real samples (tea) spiked by the various concentrations of Al (III) ion (30, 100 and 200 ppm): (a) anthocyanin solution and (b) BCNF‐ANT sensor.

Iranian people commonly consume tea, so control of aluminium contamination is critical. Based on similar determinations of Al(III) concentration in different foods, this study aims to study the application of the proposed method in real samples, and we have used the black tea (Golestan) sample for analysis. After the tea sample was prepared according to the method mentioned in Section 2, Al(III) solutions with different concentrations were added to the sample so that the final Al(III) concentration in the sample reached 30, 100 and 200 ppm. Figure 7 shows the corresponding images. The computed Δ*E* values for the tea sample are presented in Table 1. All the Δ*E* values were higher than 5, indicating that the colour changes could be recognised by naked eye in comparison to the initial colour of the solution and film.^41^ The findings indicated that the fabricated sensor could be employed in the real sample analysis successfully.

**TABLE 1:** The Δ*E* values for the real sample analysis.

**Concentration of Al(III) (ppm)**	**30**	**100**	**200**
Δ*E* value (Solution state)	22.364 ± 0.87	26.287 ± 0.73	31.934 ± 1.03
Δ*E* value (Solid state)	23.627 ± 0.94	27.298 ± 0.96	33.176 ± 0.94

Lately proved that colorimetric detection systems have been effective, easy‐to‐use and inexpensive detecting tools for metal ions. The anthocyanin extract can also be applied as an indicator for Al(III) ions with excellent selectivity, sensitivity and environmental friendliness.^41^

It was demonstrated in this study that anthocyanins from purple onion peels could be utilised as a solution‐state and portable solid‐state sensor for the detecting of Al(III) ions with a detection limit as low as 30 ppm and as high as 200 ppm in solution state, also as low as 20 ppm and as high as 300 ppm in film state.

In Table 2, the detection limit (LOD) of the fabricated sensor in this research is compared with some existing measurement systems for Al(III) detection. However, those indicators are either expensive, unselective, slow, non‐portable, non‐biodegradable, unreachable by simple preparation approaches or difficult to operate under mild conditions. Compared to previously reported sensors, low cost, no electronic components, trained personnel or sophisticated equipment were needed to apply the colorimetric sensor developed in this research.

**TABLE 2:** Comparison of previously reported Al(III) detection sensors with the BCNF‐ANT.

Sensor	Method of detection	LOD (ppm)	Reference
Pyridine‐derived Schiff‐bases	Fluorescence	0.0008	42
(E)−2‐((2‐hydroxybenzylidiene) amino)−5‐nitrophenol (HBAN)	Fluorescence	0.0003	43
Single‐armed salamo	Fluorescence	0.002	44
7‐methoxy acetylcoumarin isonicotinohydrazone	Fluorescence	0.001	3
3‐acetylcoumarinisonicotinohydrazone	Fluorescence	0.003	45
BCNF‑ANT	Colorimetric	20	This study

## 4 CONCLUSIONS

We fabricated a simple, rapid, low‐cost, selective, sensitive and portable colorimetric metallochromic sensor in the solution and solid state for the Al(III) ions detection.

The detecting process was principally based on anthocyanin extracted from purple onion peel as a spectroscopic colorimetric factor that in solid state immobilised onto a BCNFs host. In solution state, we depicted a sensor for Al(III) ions in aqueous matrices in the pH range from 6.0 to 10.0, with the capability to produce a visual colour change from pink to light green and dark green depending on Al(III) concentration. Additionally, BCNF‑ANT film can act as a sensor for Al(III) ions in the pH range of 4.0–10.0. Neutral pH was selected from the standpoint of high selectivity.

The developed sensor exhibited high selectivity in presence of the different metal ions, which include K^+^, Mn^2+^, Cu^2+^, Hg^2+^, Cr^3+^, Pb^2+^ and Ni^2+^. Anthocyanin solution and BCNF‑ANT sheet was employed in the real tea sample successfully. The results also clarified that the various foreign ions did not significantly interfere with Al(III) ions detection at optimum conditions. Al(III) contamination in food matrices and water can be monitored on‐site easily.

## CONFLICT OF INTEREST

The authors declare that they have no known competing financial interests or personal relationships that could have appeared to influence the work reported in this paper.

## Data Availability

The data that support the findings of this study are available from the corresponding author upon reasonable request.
